# Effect of *epimedium pubescen* flavonoid on bone mineral status and bone turnover in male rats chronically exposed to cigarette smoke

**DOI:** 10.1186/1471-2474-13-105

**Published:** 2012-06-19

**Authors:** Shu-guang Gao, Ling Cheng, Kang-hua Li, Wen-He Liu, Mai Xu, Wei Jiang, Li-Cheng Wei, Fang-jie Zhang, Wen-feng Xiao, Yi-lin Xiong, Jian Tian, Chao Zeng, Jin-peng Sun, Qiang Xie, Guang-hua Lei

**Affiliations:** 1 Department of Orthopaedics, Xiangya Hospital, Central South University, No.87 Xiangya Road, Changsha 410008, Hunan, China; 2 Department of Medical Record and Information, Xiangya Hospital, Central South University, No.87 Xiangya Road, Changsha, Hunan, 410008, China; 3 Orthopaedics Institute of Central South University, Changsha, Hunan, China; 4 Department of Orthopaedics, Affiliated Hospital of Xiangnan University, Chenzhou, Hunan, China

**Keywords:** Smoking, *Epimedium pubescen* flavonoid, Bone mineral density, Bone mineral content, Bone turnover, Bone histomorphometry

## Abstract

**Background:**

*Epimedii herba* is one of the most frequently used herbs in formulas that are prescribed for the treatment of osteoporosis in China and its main constituent is *Epimedium pubescen* flavonoid (EPF). However, it is unclear whether EPF during chronic exposure to cigarette smoke may have a protective influence on the skeleton. The present study investigated the effect of EPF on bone mineral status and bone turnover in a rat model of human relatively high exposure to cigarette smoke.

**Methods:**

Fifty male Wistar rats were randomized into five groups: controls, passive smoking groups and passive smoking rats administered EPF at three dosage levels (75, 150 or 300 mg/kg/day) in drinking water for 4 months. A rat model of passive smoking was prepared by breeding male rats in a cigarette-smoking box. Bone mineral content (BMC), bone mineral density (BMD), bone turnover markers, bone histomorphometric parameters and biomechanical properties were examined.

**Results:**

Smoke exposure decreased BMC and BMD, increased bone turnover (inhibited bone formation and stimulated its resorption), affected bone histomorphometry (increased trabecular separation and osteoclast surface per bone surface; decreased trabecular bone volume, trabecular thickness, trabecular number, cortical thickness, bone formation rate and osteoblast surface per bone surface), and reduced mechanical properties. EPF supplementation during cigarette smoke exposure prevented smoke-induced changes in bone mineral status and bone turnover.

**Conclusion:**

The results suggest that EPF can prevent the adverse effects of smoke exposure on bone by stimulating bone formation and inhibiting bone turnover and bone resorption.

## Background

Recent epidemiological studies have yielded increasingly strong evidence that even low level of tobacco smoke exposure poses a risk for health, particularly affecting the lung and skeleton [1-12]. Smoking was also associated with lower areal BMD and reduced cortical thickness in young men [13]. Systemic nicotine may have a significant adverse impact on bone wound healing and inhibit the bone matrix-related gene expressions required for wound healing [14,15].

Tobacco has been identified as the cause for the death of half of its regular users, but insufficient consideration has been given to the hazard of smoking to passive smokers. Thus, a growing interest has been focused on factors that can protect from the harmful action of cigarette smoke.

*Epimedii herba* (Yinyanghuo) is one of the most frequently used herbs in formulas that are prescribed for the treatment of osteoporosis in China and its main constituent is *Epimedium pubescen* flavonoid (EPF). Recently, EPF which consist of three phytoestrogenic compounds (Icariin, Genistein and Daidzein), has been fractioned by a series of standardized extraction-isolation procedures. Studies have shown the extract of *Epimedii herba* reduces the occurrence of osteoporosis not only in experimental models [16,17] but also in clinical studies [18].

It is unclear whether EPF during chronic exposure to cigarette smoke may have a protective influence on the skeleton. Therefore, this study was designed to evaluate the effects of EPF on BMD, BMC, bone turnover, bone histomorphometry and biomechanical properties in a rat model of human relatively high exposure to cigarette smoke.

## Methods

### Animals and experimental protocol

Fifty adult 8-month-old (body weight 352 ± 24 g) male Wistar rats were purchased from SLRC Laboratory Animal (Shanghai, China). The experiment was performed with the approval of the Animal Research Committee of the Xiangya Hospital, and the animals were cared for in the Experimental Animal Center of Xiangya Hospital. During the whole experiment the animals were housed in stainless steel cages (one rat per cage) at conventional controlled conditions (temperature 25 ± 2°C, relative humidity of 50 ± 10%, 12 h light–dark cycle) and had free access to the standard laboratory food and tap water.

The rats were acclimated to conditions for one week before the experiment and randomly assigned to five groups of 10 rats each. One group drank tap water and it served as a control. The smoke-exposed groups were kept in an environment with smoking for 4 months and it served as passive smoking groups, whereas the remaining three groups were supplemented with EPF during exposure to cigarette smoke. EPF (Jiuhui pharmaceutical Corp., Hunan, China) was administered at three dosage levels (75, 150 or 300 mg/kg per day) in drinking water for 4 months and expressed as L-EPF, M-EPF, and H-EPF.

### Cigarette smoking

The rats were exposed to sidestream cigarette smoke (CS) from an ashtray which was placed 10 cm below the rat cage in an exposure box (30 cm × 5 cm × 15 cm) made of polypropylene, which was placed in a laboratory draft chamber [19]. Using this equipment, the time, amount, and interval of smoke to be sent into the chamber was defined, and 10 cigarettes could be set at a time. All blood samples were collected from the abdominal vena cava into heparinized tubes at the same time of day (9:00–10:00 AM) and centrifuged at 1500 r.p.m. and 4°C for 5 min. Separated plasma was stored at −70°C for subsequent determination of blood nicotine concentrations. The blood concentration of nicotine was measured to reflect the intensity of exposure to smoke. Cigarette smoke was sent for 10 min and then the box was ventilated with room air for 10 min. This procedure was repeated 10 times at 1-h intervals such that the amount of cigarette smoking per day in the rats was equal to in heavy smokers who smoke equal to 30 cigarettes per day [20]. The impact of passive smoking was evaluated by measuring blood nicotine concentrations [21].

### The length of femur and height of the lumbar

The length of the femur and the posterior height and anterior height of the L4 were measured with a precision calliper (±0.02 mm; China Machine Building International Corporation Hunan Co., Hunan, China). These measurements were made in duplicate by the same examiner. The results of the length of the femur were expressed as the mean value of both femurs.

### Bone densitometry

The bone mineral content (BMC) and density (BMD) at femur neck and lumbar vertebrae (L4-L6) were measured ex-vivo by dual-energy X-ray absorptiometry (DXA; Hologic QDR-4500A) equipped with appropriate software for bone assessment in small animals [22]. The scan resolution was 1.0 × 1.0 mm and scan speed was 10 mm/s. The measurements with repositioning of the bones were repeated for three times to calculate the Coefficients of variation (CV). The CV for BMC and BMD on femur neck in our laboratory were 2.1% and 2.2%, and on L4-L6 were 0.9% and 0.8%, respectively. The results of the BMC or BMD in femur neck were expressed as the mean value of both femurs. Similarly to the femur neck, the results of the BMC or BMD in lumbar spine were expressed as the mean value of L4-L6.

### Biomarkers of bone turnover

Serum osteocalcin concentration was measured by an enzyme-linked immunosorbent assay (Rat Mid^TM^ Osteocalcin ELISA kit, IDS Inc., Fountain Hills, AZ, USA) according to the manufacturer's instructions. The intra- and inter-assay variations of this measurement in our laboratory were 6.8% and 8.9%, respectively. Serum b-ALP was measured by immunoassay using the Access Ostase Assay (Beckman Access, Beckman Coulter Inc., Fullerton, CA, USA). The intra-assay and interassay coefficients of variation were 4.1% and 5.2%, respectively. Serum TRACP 5b level was measured by a solid phase immunofixed enzyme activity assay (Rat TRACP^TM^ Assay, IDS Inc., Fountain Hills, AZ, USA) according to the manufacturer's instructions to assess bone resorption. The intra- and inter-assay variations of this measurement in our laboratory were 4.9% and 7.3%, respectively.

### Bone histomorphometry

The one-third proximal segment of left tibia was embedded in methyl methacrylate (MMA) after Villanueva’s staining. A 10-μm-thick mid-sagittal section was obtained using a microtome (MR2065; Reichert-Jung, Heidelberg, Germany). Histomorphometry variables were analyzed in both trabecular bone and cortical bone using an image-analyzing computer (Cosmozone 1 S; Nikon, Tokyo, Japan) linked to a microscope. The parameters measured included trabecular bone volume, trabecular number, trabecular thickness, cortical thickness, BFR/BS (bone formation rate), trabecular separation, osteoclast surface per bone surface and osteoblast surface per bone surface. Standard bone histomorphometrical nomenclatures, symbols, and units were used as described in the report of the American Society of Bone and Mineral Research Histomorphometry Nomenclature Committee [23]. The sections were also carefully polished, etched with a solution of HCl 0.1 N for 60 s, and gold palladium-coated for backscattered scanning electron microscopic (SEM) analysis.

### Bone biomechanical testing

Biomechanical properties were estimated in a compression test of the L4 performed by AG-IS Universal Testing Instrument (Shanghai, Shimadzu, Japan) [21]. The L4 was compressed until failure along its longitudinal axis at a displacement rate of 2 mm/min using the flat-tipped pin 4.5 mm in diameter. Load–displacement curves were recorded on-line and analyzed for the following bone biomechanical properties: yield load (the force causing the first bone damage), ultimate load (the force at which fracture occurs), displacement at yield (maximal bone deformation under elastic conditions) and at ultimate (bone deformation at the fracture point), stiffness (the slope of the linear, elastic part of the load–displacement curve), and work to the L4 failure.

### Statistical analysis

Results of all measurements were presented as mean ± S.D. for ten rats in each group. A one-way analysis of variance (ANOVA) was first performed to determine whether there were statistically significant (*p* < 0.05) differences among the experimental groups. Further, the Duncan’s multiple range post-hoc test was used for comparisons between individual groups and to determine which means differed statistically significantly (*p* < 0.05).

## Results

### Body weight, blood nicotine concentrations, femur length and L4 height

The rats were weighed weekly throughout the whole experiment. The initial weights and final weights are shown in Table1. At the beginning of the study and during the 4-month experimental period there were no statistically significant differences in the body weight of rats among the five groups (*P* > 0.05). At termination, the body weight of rats receiving passive smoking or/and EPF was similar to that noted in the control group reaching 362.3 ± 22.2 g. The mean water consumption was 11.5 ±1.8 ml/100 g body weight/day. Total food and water supply in smoke-exposed groups were similar to that in the control groups.

**Table 1:** Body weight, femur length and L4 height

** *Group* **	** *Control* **	** *Smoke* **	** *Smoke + L-EPF* **	** *Smoke + M-EPF* **	** *Smoke + H-EPF* **
Initial weights(g)	353 ± 23	351 ± 28	351 ± 16	355 ± 25	352 ± 17
Final weights(g)	362 ± 22	360 ± 26	358 ± 27	368 ± 29	364 ± 28
Length of femur (mm)	39.85 ± 0.54	39.73 ± 0.46	39.18 ± 0.71	39.88 ± 0.59	39.57 ± 0.66
Anterior height of L4 (mm)	7.32 ± 0.28	7.30 ± 0.24	7.35 ± 0.29	7.33 ±0.30	7.31 ±0.26
Posterior height of L4 (mm)	7.63 ± 0.38	7.62 ± 0.35	7.68 ± 0.34	7.65 ± 0.29	7.70 ±0.41

Values are means (for 10 rats) with their standard deviation.

The concentrations in the controls and the 4-month smoke-exposed rats were 2.4 ± 1.3 ng/ml and 40.6 ± 5.9 ng/ml, respectively. The blood nicotine concentrations in the 75, 150 and 300 mg/kg/d EPF groups receiving passive smoking were 39.2 ± 3.7 ng/ml, 40.7 ± 3.5 ng/ml and 41.2 ± 3.9 ng/ml, respectively. The blood nicotine concentration in the rats receiving passive smoking or/and EPF was significantly higher than those in controls (*P* < 0.05).

The femur length and the anterior height and posterior height of L4 in the control rats were 39.85 ± 0.54 mm, 7.32 ± 0.28 mm and 7.63 ± 0.38 mm, respectively, after 4 months of the experiment. The femur length and L4 height were unchanged by any treatment (Table1).

### Bone densitometry

**Figure 1:**
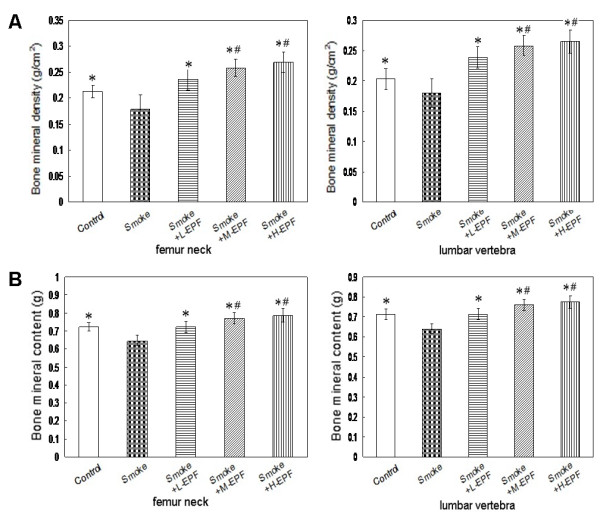
**BMD (A) and BMC (B) at the femur neck and lumbar vertebra.** BMD and BMC at the lumbar vertebrae and femur in the 4-month smoke-exposed rats were significantly lower than that in other groups. Significantly higher BMD and BMC at the femur neck and lumbar vertebra were founded in the 150 and 300 mg/kg/d EPF group receiving passive smoking compared to the control group. Mean ± SD (n = 10 in each group). *P < 0.05 compared to smoke-exposed rats; ^#^*P* < 0.05 compared to controls.

BMD and BMC of the femur neck and lumbar vertebrae are demonstrated in Figure1. BMD and BMC of the femur neck and lumbar vertebrae were lower (*P* < 0.05) in the smoke-exposed rats compared to control groups after 4 months of the experiment. There was no significant difference in the BMD and BMC at the femur neck and lumbar vertebrae between the controls and 75 mg/kg/day EPF groups receiving passive smoking. The 150 or 300 mg/kg/day EPF groups receiving passive smoking had greater BMD and BMC at the femur neck and lumbar vertebrae than control groups.

### Bone turnover

**Figure 2:**
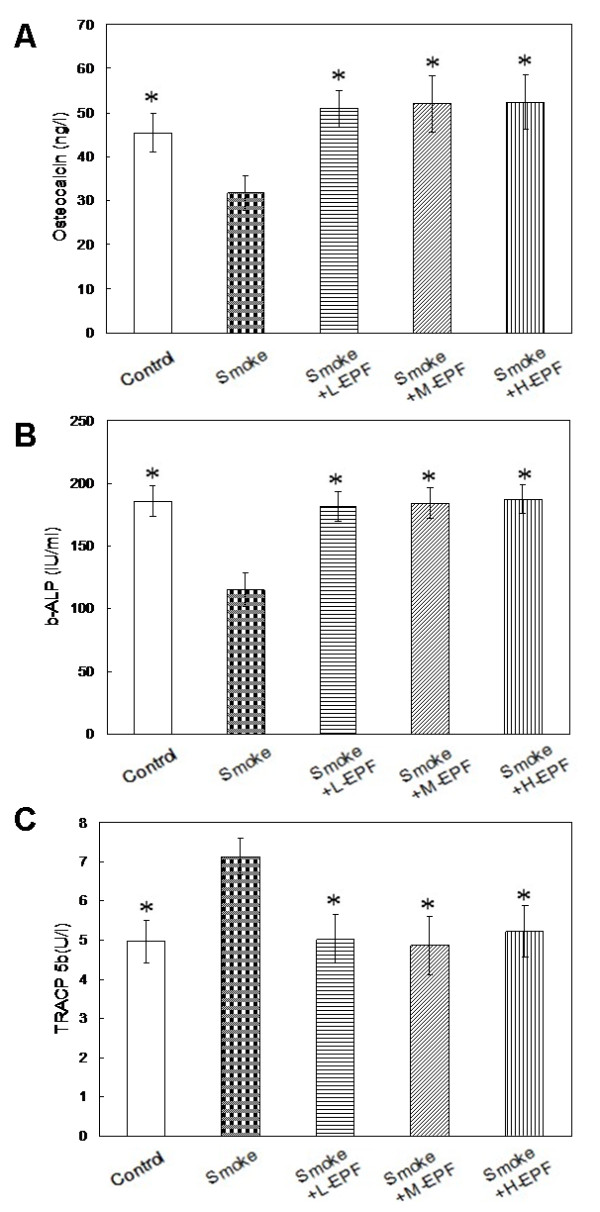
**Levels of osteocalcin (A), b-ALP (B) and TRACP 5b (C) in five groups.** The smoke-exposed male rats had lower osteocalcin concentration and b-ALP activity and higher TRACP 5b levels than the controls. Mean ± SD (n = 10 in each group). *P < 0.05 compared to smoke-exposed rats.

Figure2 shows the results of the bone turnover measurements. Significantly lower osteocalcin concentration and b-ALP activity and higher TRACP 5b levels were found in the smoke-exposed male rats compared to controls. There was no significant difference in serum osteocalcin concentration and b-ALP activity and TRACP 5b levels among the controls, 75, 150, and 300 mg/kg/d EPF groups receiving passive smoking (*P* > 0.05) (Figure2).

### SEM and histomorphometric findings

**Figure 3:**
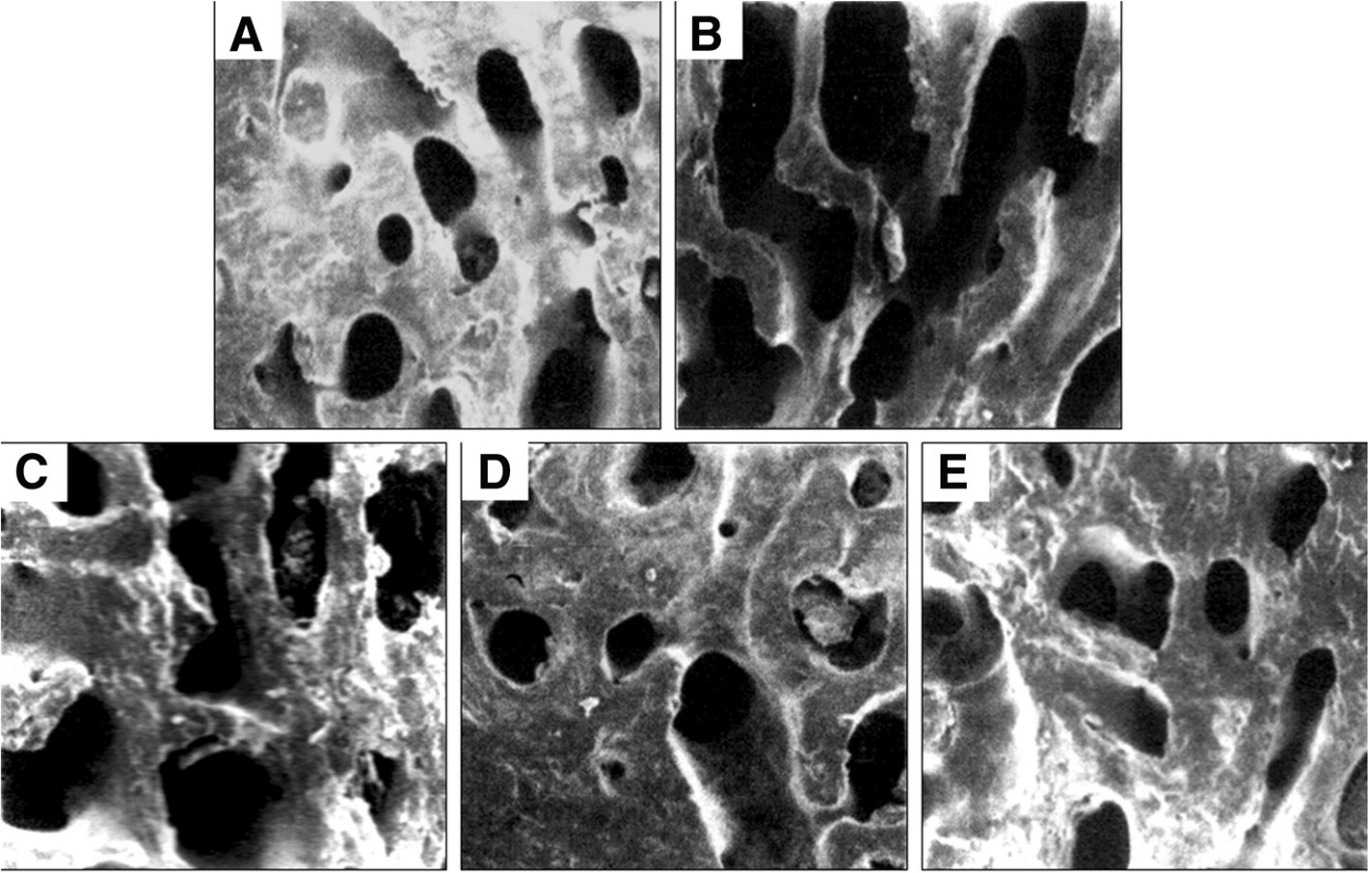
**SEM micrographs of the tibia.** The rats of the control group revealed normal compactness of the diaphysis and competent trabeculae (**A**). The passive smoking animals showed sparse, uniform thinning of the trabeculae resulting in widened intertrabecular spaces (**B**). The passive smoking and L-EPF group exhibited significant remodeling progress with mineralization (**C**). The group treated with M-EPF (**D**) and H-EPF (**E**) showed nearly essential features of normal bone.

Figure3 displays five images obtained by SEM examination of the tibia. The rats of the control group revealed normal compactness of the diaphysis and competent trabeculae (Figure3A). The passive smoking animals showed sparse, uniform thinning of the trabeculae resulting in widened intertrabecular spaces (Figure3B). The passive smoking and L-EPF group exhibited significant remodeling progress with mineralization (Figure3C). The group treated with M-EPF (Figure3D) and H-EPF (Figure3E) showed nearly essential features of normal bone.

**Figure 4:**
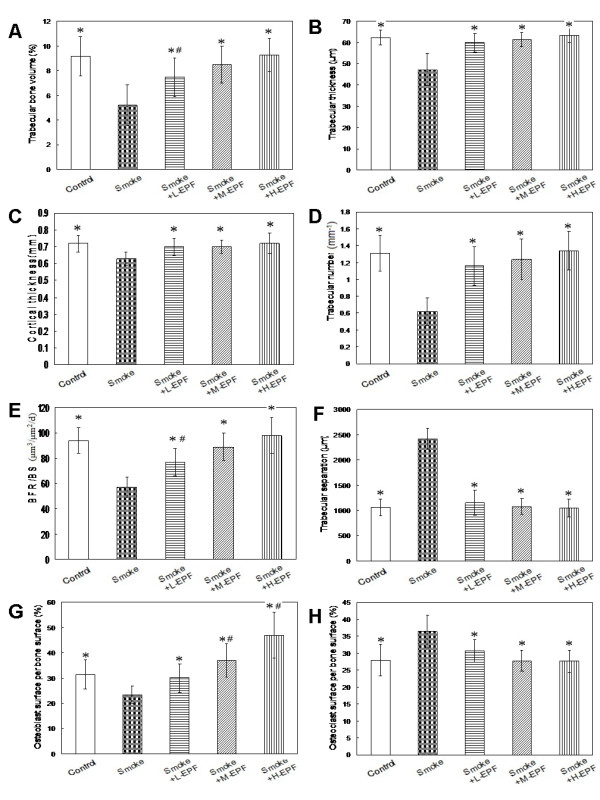
**Histomorphometric parameters of tibia.** Significantly greater trabecular separation (**F**) and osteoclast surface per bone surface (**H**), lower trabecular bone volume (**A**), trabecular thickness (**B**), trabecular number (**C**), cortical thickness (**D**), BFR/BS (**E**), osteoblast surface per bone surface (**G**) were noted in the smoke-exposed male rats compared to controls. EPF returned the histomorphometric parameters to control levels. Mean ± SD (n = 10 in each group). *P < 0.05 compared to smoke-exposed rats; ^#^*P* < 0.05 compared to controls.

Results concerning bone histomorphometry of the tibia are summarized in Figure4. In the smoke-exposed rats, trabecular bone volume, trabecular thickness, cortical thickness, trabecular number, BFR/BS and osteoblast surface per bone surface were lower (by 43.5%, 24.2%, 12.5%, 52.7%, 39.4% and 25.4%, respectively), whereas trabecular separation and osteoclast surface per bone surface were greater (by 128.8 and 30.6%, respectively) compared to the control group. Compared to control group, lower trabecular bone volume and BFR/BS (bone formation rate) were found in the 75 mg/kg/d EPF group receiving passive smoking. No differences were noted in the other bone histomorphometry measurements between this group and the control group. In the 150 and 300 mg/kg/d EPF group receiving passive smoking, greater osteoblast surface per bone surface was noted, but there was no significant difference in the other bone histomorphometry measurements, when compared to the control group.

### Biomechanical properties

The biomechanical properties of L4 are shown in Table2. The bone yield load, ultimate load, displacement at yield, displacement at ultimate, stiffness and work to failure were, respectively 23.8%, 26.5%, 9.2%, 21.9%, 21.8% and 29.1% lower in smoke-exposed compared to control rats. No significant difference of the biomechanical properties of L4 was noted among the controls, 75, 150, and 300 mg/kg/d EPF groups receiving passive smoking.

**Table 2:** Biomechanical properties of L4 in five groups

** *Group* **	** *Yield load (N)* **	** *Ultimate load (N)* **	** *Displacement at yield (mm)* **	** *Displacement at ultimate (mm)* **	** *Stiffness (N/mm)* **	** *Work to failure (J)* **
Control	126.24 ± 8.01*	235.1 ± 38.6*	0.791 ± 0.051*	1.888 ± 0.245*	254.0 ± 64.4*	0.491 ± 0.045*
Smoke	96.24 ± 14.67	172.9 ± 15.9	0.721 ± 0.043	1.475 ± 0.136	198.6 ± 42.3	0.398 ± 0.054
Smoke + L-EPF	117.43 ± 17.09*	225.1 ± 38.6*	0.782 ± 0.052*	1.723 ± 0.167*	238.2 ± 54.7*	0.466 ± 0.039*
Smoke + M-EPF	115.52 ± 15.12*	238.1 ± 36.2*	0.786 ± 0.058*	1.779 ± 0.138*	248.2 ± 46.2*	0.465 ± 0.077*
Smoke + H-EPF	128.52 ± 16.39*	245.8 ± 38.2*	0.795 ± 0.060*	1.783 ± 0.196*	258.2 ± 35.5*	0.488 ± 0.063*

Mean ± SD (n = 10 in each group).

*P < 0.05 compared to smoke-exposed rats.

## Discussion

The damaging action of chronic smoke exposure on the skeleton and its possible mechanisms have been extensively studied by us and reported in details [24-31]. We demonstrated that the BMD and BMC of the femur neck and lumbar vertebrae were lower in 4-month smoke exposed rats than in controls. Moreover, lower serum osteocalcin concentration and activity of b-ALP and greater TRACP 5b level were noted in the passive smoking group, consistent with the results of previous report [21,28,29]. These results indicated that smoke exposure suppressed bone formation and increased bone resorption. In addition, significant lower trabecular bone volume, trabecular thickness, trabecular number, cortical thickness, bone formation rate and osteoblast surface per bone surface and significant greater trabecular separation and osteoclast surface per bone surface were found in the passive smoking groups. Taking into account the observations made in the present study and the previous reports [24,27,31] the damaging action of smoke exposure on the skeleton might result from its direct action. This mechanism involved stimulation of bone resorption and inhibition of its formation through a direct influence on both differentiation and activity of osteoblastic and osteoclastic bone cells.

Chinese herbal medicine has been widely used for thousands of years in the treatment of fracture and joint diseases. EPF was found to have a preventive effect on osteoporosis by ovariectomy rat models [16,32] and exert a beneficial effect on preventing bone loss for postmenopausal women [33]. Icariin is a major constituent of flavonoids isolated from the *herb Epimedium*. The previous studies reported that icariin can stimulate proliferation and differentiation of human osteoblasts by increasing production of BMP-2, BMP-4, NO synthesis, promoting the ALP activity and type I collagen expression, subsequently regulating Cbfa1/Runx2, OPG, and RANKL gene expressions [17,34-36]. Meanwhile, icariin also was found to can inhibit osteoclast formation by RANKL and macrophage-colony stimulating factor, and inhibit osteoclast differentiation and bone resorption by suppression of MAPKs/NF-κB regulated HIF-1α and PGE(2) synthesis[37,38].

However, the beneficial influence of EPF on the skeleton at the exposure to cigarette smoke has not been shown until now. The enhanced serum osteocalcin concentration and b-ALP activity due to EPF indicated an enhanced osteoblastic activity in the whole skeleton. Our findings about TRACP 5b, would indicate that EPF can prevent the smoke-induced increased osteoclast activities. The pattern of change in bone turnover was consistent with the findings from previously published study on EPF in an ovariectomized rat model [39]. Icariin, one of the major flavonoids in EPF (77%), has been reported to stimulate proliferation of rat bone marrow stromal cells and increase the number of colony unit forming-fibroblasts staining positive for alkaline phosphatase in a dose-dependent manner, whereas the alkaline phosphatase activity, OC secretion, and calcium deposition level of rat bone marrow stromal cells were also increased by icariin in a dose-dependent manner, suggesting a potential anabolic effect of icariin on bone [40]. Furthermore, Genistein and Daidzein, the other two flavonoids within EPFs (23%), have also been reported to have a stimulatory effect on protein synthesis and on alkaline phosphatase release by various types of osteoblast cells in vitro [41-43]. Thus, the beneficial effect of EPF consisted in the stimulation of osteoblast activity as well as the reduction of smoke-induced bone resorption.

Kim BS et al. [44] showed that nicotine suppressed osteoblast proliferation and inhibited the expression of some key osteogenic (TGF-β1, BMP-2) and angiogenic mediators (PDGF-AA, VEGF) in the in vitro experimental model. In addition, Ma L et al. [45] reported that Calcium accumulation, ALP activity, and mRNA levels of ALP, bone sialoprotein (BSP), collagen type I α 1 (Col1αI), and runt-related transcription factor 2 (Runx2) were significantly decreased by treatment with nicotine, while osteocalcin transcripts decreased by treatment with nicotine. Thus, nicotine may interact with EPF since nicotine also regulates proliferation and differentiation of human osteoblasts and modulates bone metabolism-associated gene expression in osteoblast cells [44,46].

In this study, the 8-month rats were in the final phase of bone mass formation (bone mass consolidation) and its metabolic processes in the bone slow down. The cellular mechanism behind the anabolic effect of EPF has not been established clearly yet. EPF-treatment may induce a positive bone balance in the remodeling cycle, but this has not been shown directly by histomorphometry. Alternatively, part of the bone anabolic effect of icariin or other EPF components may result from the induction of bone formation by modelling. Such an effect has been demonstrated already to be part of the bone anabolic action of parathyroid hormone by Lindsay and Dempster in iliac crest biopsies [47], and the same may be true for icariin. Our study showed that EPF can succeed to bring the histomorphometric parameters and biomechanical properties back to control levels and prevent from all of the adverse effects of smoke exposure on bone. This study also showed that EPF can exhibit a beneficial effect on preventing bone loss in passive smoking rats, which was shown by maintained BMD and BMC at the femur neck and lumbar vertebrae in the EPF treatment group compared with significantly decreased BMD and BMC in the passive smoking group. In addition, it is important to note that the higher mean values of the densitometric parameters were found in the EPF group receiving passive smoking than in the passive smoking group or the control group. The alternative explanations for the increase in BMD may include induction of periosteal and endocortical bone modeling (increased cortical thickness), induction of trabecular micro modeling and prevention of marrow cavity expansion, etc. These results may suggest that EPF via its positive effect on bone might also somewhat induce bone modeling.

The influence of EPF on the bone mineral status and bone turnover during the exposure to cigarette smoke somewhat differed depending on EPF dose. Additional daily consumption of 75 mg EPF/kg body wt was noted to be sufficient in this regard; however, the daily dose of 150 or 300 mg EPF/kg body wt may offer stronger protection. The osteoblast surface and many static parameters of bone geometry indicated that the bone formation was dose dependent. The results suggested that there was a bone formation inducing component in EPF which induced a bone anabolic response independent of its effect on bone resorption. As mentioned above this could be in the form of induction of bone modeling, which was independent of bone resorption.

The effects of EPF on bone have been reported [16]. Thus, this study has not a series of rats treated only with EPF (without smoking). Considering how this medication could be used in the future, it would be necessary to include an additional control group that is kept under normal environment without smoke but receives EPF. That is exactly what we are planning to do in the near future.

Though the present study demonstrate that EPF has a protective effect on the skeleton in smoke-exposed rats, it is not clear whether EPF has a protective effect on the cardio-respiratory effects of smoking and the increased risk of cancer up to now. Even if the smoker can inhibit their bone loss by taking this medicine, smoking also is not ok. According the 2004 Surgeon's General Report, every smoker lives on average 13–14 years less than non-smokers. Smoking also causes cancer, damages organs, and weakens the immune system. In addition, smoking can also harm those nearby, in terms of second-hand smoke. Therefore, it would still be recommended to quit smoking or never initiate it in the first place.

## Conclusion

In summary, the present study, in a male rat model of relatively high human exposure to cigarette smoke, suggests that EPF supplementation during chronic exposure to cigarette smoke can prevent from smoke-induced bone loss by stimulating bone formation and inhibiting bone turnover and bone resorption, without resulting in a detectable effect on the bone.

## Competing interests

The author(s) declare that they have no competing interests.

## Authors’ contributions

SGG, LC and GHL were involved in the conceptual discussion and design of the study. WHL and SGG carried out the biochemical analysis. MX, WJ and LCW performed the bone densitometry analysis. FJZ, WFX and YLX performed the bone biomechanical testing. TJ, CZ JPS and QX carried out the bone histomorphometric analysis. SGG and GHL drafted the manuscript. KHL and GHL obtained the funding for National Natural Science Foundation of China. All authors read and approved the final manuscript.

## Pre-publication history

The pre-publication history for this paper can be accessed here:

http://www.biomedcentral.com/1471-2474/13/105/prepub
